# Exploiting Meltable Protein Hydrogels to Encapsulate and Culture Cells in 3D

**DOI:** 10.1002/mabi.202200134

**Published:** 2022-07-13

**Authors:** Gema Dura, Maria Crespo‐Cuadrado, Helen Waller, Daniel T. Peters, Ana Ferreira‐Duarte, Jeremy H. Lakey, David A. Fulton

**Affiliations:** ^1^ Chemical Nanoscience Laboratory Chemistry‐School of Natural and Environmental Sciences Newcastle University Newcastle upon Tyne NE1 7RU UK; ^2^ School of Engineering Stephenson Building Newcastle University Newcastle upon Tyne NE1 7RU UK; ^3^ Institute for Cell and Molecular Biosciences Medical School Newcastle University Newcastle upon Tyne NE1 7RU UK; ^4^ Departamento de Química Inorgánica Orgánica y Bioquímica Universidad de Castilla‐La Mancha Facultad de Ciencias yTecnologías Químicas‐IRICA Avda. C. J. Cela, 10 Ciudad Real 13071 Spain

**Keywords:** bacterial fimbriae, bioorthogonal, capsular antigen fragment 1, crosslinked hydrogel, protein polymer

## Abstract

There is a growing realization that 3D cell culture better mimics complex in vivo environments than 2D, lessening aberrant cellular behaviors and ultimately improving the outcomes of experiments. Chemically crosslinked hydrogels which imitate natural extracellular matrix (ECM) are proven cell culture platforms, but the encapsulation of cells within these hydrogel networks requires bioorthogonal crosslinking chemistries which can be cytotoxic, synthetically demanding, and costly. Capsular antigen fragment 1 (Caf1) is a bacterial, polymeric, fimbrial protein which can be genetically engineered to imitate ECM. Furthermore, it can, reversibly, thermally interconvert between its polymeric and monomeric forms even when chemically crosslinked within a hydrogel network. It is demonstrated that this meltable feature of Caf1 hydrogels can be utilized to encapsulate neonatal human dermal fibroblasts at a range of cell densities (2 × 10^5^–2 × 10^6^ cells mL^−1^ of hydrogel) avoiding issues with chemical cytotoxicity. These hydrogels supported cell 3D culture for up to 21 d, successfully inducing cellular functions such as proliferation and migration. This work is significant because it further highlights the potential of simple, robust, Caf1‐based hydrogels as a cell culture platform.

## Graphical abstract

Just melt, cool and add cells! Cells are encapsulated within a chemically crosslinked hydrogel that exploits the meltable feature of the protein polymer capsular antigen fragment 1. This approach avoids the need for potentially cytotoxic or costly chemical crosslinking chemistries and can be used to encapsulate even relatively high cell densities.

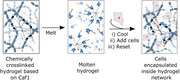

## 1 Introduction

In vitro cell culture is important in a wide range of biomedical applications including regenerative medicine,^[^ ^1^, ^2^, ^3^ ^]^ disease modeling,^[^ ^4^, ^5^, ^6^ ^]^ personalized therapies,^[^ ^7^, ^8^ ^]^ and drug testing/discovery.^[^ ^9^, ^10^ ^]^ Cell culture and cell‐based assays are usually performed using 2D layers of cells on flat plastic or glass surfaces. Although experimentally simple and relatively low‐cost, 2D cell culture does not mimic the native in vivo cellular microenvironment. Consequently, cells cultured in 2D lack the cell–cell and cell–matrix interactions found in 3D microenvironments and can display aberrant cell behaviors including changes in cell morphology, polarity, and method of division.^[^ ^11^ ^]^

In the recent years there has been considerable effort to develop 3D cell culture models that by bridging the gap between 2D models and in vivo animal models better reflect cell microenvironments.^[^ ^12^, ^13^ ^]^ Of particular interest is the 3D culture of cells within hydrogel matrices.^[^ ^14^ ^]^ Hydrogels make appealing cell culture materials as they can be engineered to provide suitable microenvironments for cell adhesion, proliferation, and migration, and promote the exchange of nutrients and signaling molecules.^[^ ^15^, ^16^, ^17^, ^18^ ^]^

Hydrogels fall into one of two broad classifications—physical or chemical—which indicate the nature of the bonding which maintain the structure of the hydrogel matrix. Physical hydrogels are often protein or peptide‐based and offer the advantage that no reactive functional groups and crosslinking chemistries are required to drive gelation, making 3D cell encapsulation straightforward. Physical hydrogels also are associated with shear‐thinning behaviors which can be advantageous for injection. However, on account of the weak interactions involved in physical hydrogels, they often suffer from poor mechanical properties.^[^ ^19^ ^]^ Furthermore, the mechanical properties of physical hydrogels can be difficult to tune since it can be difficult to alter crosslinking densities, and it is also difficult to control the degradability of physical hydrogels. In this regard, chemically crosslinked hydrogels are more appealing than physically crosslinked gels as they possess enhanced stability under physiological conditions, potential for tunable degradation and there is scope to manipulate their mechanical properties by control of crosslinking.^[^ ^20^ ^]^

A key step in 3D cell culture is the encapsulation of the cells within the chemically crosslinked hydrogel in a way that ensures excellent levels of cell viability. These requirements have led to the development of a wide range of bioorthogonal crosslinking chemistries including photopolymerization,^[^ ^21^, ^22^ ^]^ enzyme‐induced crosslinking^[^ ^23^ ^]^ and click chemistry reactions such as oxime formation and Schiff base formation.^[^ ^24^ ^]^ However, the coupling agents, catalysts or photoinitiators often used to facilitate crosslinking introduce potentially cytotoxic small molecules, affecting the biocompatibility of the hydrogel. To circumvent these issues, crosslinking chemistries with improved biocompatibility have been developed such as Diels‐Alder,^[^ ^25^, ^26^ ^]^ Michael additions,^[^ ^27^ ^]^ and strain‐promoted azide‐alkyne cycloadditions,^[^ ^28^, ^29^ ^]^ but these approaches can introduce extra synthetic complexity and cost into the cell encapsulation process. With increasing demand for sophisticated cell culture, there is a need for improved bioorthogonal crosslinking strategies that avoid cytotoxicity whilst avoiding the need for time‐consuming and expensive synthetic organic chemistry.

**Figure 1:**
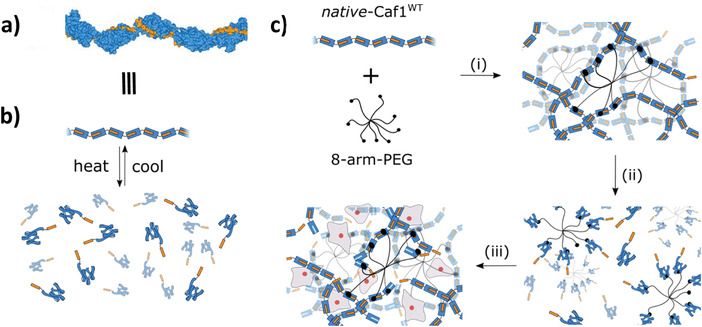
a) Model of a segment of Caf1 polymer (generated from PDB entry 1P5U). The N‐terminal donor strands (colored orange) are complexed by the acceptor domains (colored blue) of adjacent subunits. Caf1 is a polymer of immunoglobulin‐like domains and as such it resembles the predominant family of extracellular proteins in humans which includes fibronectin type III repeats.^[^ ^29^ ^]^ b) Cartoon depicting the reversible thermal unfolding of Caf1 polymers. When heated above the subunit melting temperature (*T*_m_ > 86 °C), the subunits thermally unfold with concomitant decomplexation of the N‐terminal donor strands, causing Caf1 to depolymerize into its monomeric form. When the subunits are incubated at room temperature they subsequently refold with donor strand recomplexation and concomitant repolymerization. c) The encapsulation of live cells inside Caf1 hydrogels without the addition of reactive crosslinkers. i) The chemical crosslinking of *native*‐Caf1^WT^ with a 40 kDa *N*‐hydroxysuccinamide(NHS)‐activated ester functionalized multiarm PEG (polyethylene glycol) affords a soft chemically crosslinked hydrogel. Crosslinking is driven by the reaction of lysine residues upon Caf1 with the NHS‐esters of the multiarm PEG. On account of the strong kinetically inert noncovalent interactions between the Caf1 subunits, the resulting hydrogel network is best described as a chemical network. ii) When heated above *T*_m_ the Caf1 subunits unfold causing the hydrogel to melt. iii) The molten hydrogel was cooled and immediately seeded with live cells. As the Caf1 subunits refold the hydrogel network reforms, homogenously encapsulating the cells. As the donor–acceptor interaction is unique to Caf1, the encapsulation process can be considered to be bioorthogonal in nature.

In this work, we demonstrate how the meltable feature of hydrogels based upon the bacterial fimbriae Capsular antigen fragment 1 (Caf1) can be successfully utilized to encapsulate live cells within a chemically crosslinked 3D hydrogel matrix. Caf1 (**Figure** **1**a) is an IgSF (Immunoglobulin Superfamily) protein polymer which forms a protective “nonstick” surface coating around the plague bacterium *Yersinia pestis*.^[^ ^30^ ^]^ The polymer is formed from monomeric 15 kDa protein subunits that donate a single *β*‐strand to the next monomer in the chain, linking together the subunits through exceptionally strong and kinetically inert noncovalent interactions.^[^ ^31^ ^]^ The *K*_A_ of the subunit interaction is estimated to be at least 10^14^ m ^−1^ with dissociation times in the order of billions of years.^[^ ^32^, ^33^, ^34^ ^]^ These features are highly unusual for a host–guest complex, which are usually less tight and far more dynamic in nature, and thus the noncovalent interactions between Caf1 subunits can be considered to possess kinetic stability that is more on par with that of strong covalent bonds. Despite the strong and stable nature of the intersubunit interactions, we recently discovered that Caf1 demonstrates a meltable behavior as it can be repeatedly recycled between its polymeric and monomeric states by changes in temperature (Figure 1b).^[^ ^35^ ^]^ This meltable feature is also displayed by Caf1 hydrogels which show full, repeatable, but delayed, repolymerization upon cooling. Previously, we showed (Figure 1c) how this feature could be used to trap live cells inside an unreactive pre‐crosslinked Caf1 network. Here, we exploit this discovery to explore in detail how the meltable feature of Caf1‐based hydrogels can be utilized in 3D cell culture, using encapsulated human dermal fibroblasts (hDFbs) as a model cell line. We show that it is possible to successfully encapsulate a range of cell densities, and even relatively high cell densities (2 × 10^6^ cells mL^−1^) do not interfere with the reformation of the Caf1‐hydrogel. We also demonstrate how it is possible to control the RGDS density within the hydrogel and how this feature can impact on cell behavior over long time periods (up to 21 d). We also benchmark cell encapsulation in Caf1 hydrogels against Geltrex, a commercially available hydrogel extracted from murine Engelbreth–Holm–Swarm (EHS), where we find Caf1 hydrogels better promote metabolic activity. The successful outcomes of this study, together with advantageous features of Caf1 including its animal‐free production, absence of batch‐to‐batch variability and the ability to fine tune its bioactivity, suggest that Caf1‐based hydrogels possess considerable potential as a versatile platform for 3D cell culture.

## 2 Results and Discussion

### 2.1 Production of Caf1‐PEG Hydrogels

In this study, two variants of Caf1 were used: the wild‐type protein (*native*‐Caf1^WT^) and an engineered version (*native*‐Caf1^RGDS^) where a single integrin binding motif, RGDS (Arg‐Gly‐Asp‐Ser), has been inserted into loop 5 of the subunit. The prefix *native*‐ simply refers to bacteria‐expressed polymers. We have previously demonstrated^[^ ^30^ ^]^ that Caf1^WT^ displays a “nonstick” behavior with cells, whereas the insertion of the RGDS motif reverses this behavior, providing an anchor point for cell‐surface integrins. We envisaged that hydrogels incorporating Caf1^RGDS^ subunits would thus encourage encapsulated cells to grow and proliferate, whereas hydrogels based solely upon Caf1^WT^ would not.

**Figure 2:**
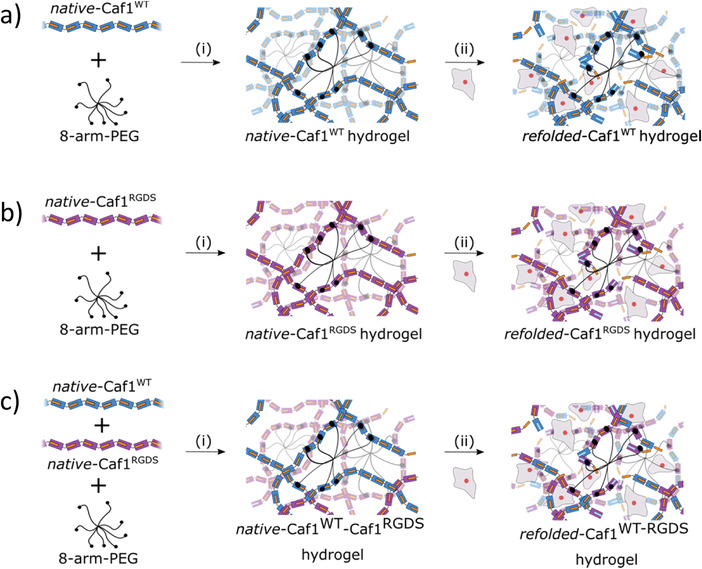
The production of Caf1 hydrogels and encapsulation of live cells inside the hydrogel network. a) Step (i). *Native*‐Caf1^WT^ was reacted with 8‐arm PEG to form *native*‐Caf1^WT^ hydrogel. Step (ii). This hydrogel was melted, immediately cooled to room temperature and a suspension of hDFb cells added. After cooling, *refolded*‐Caf1^WT^ was formed encapsulating hDFbs. b) Step (i). *Native*‐Caf1^RDGS^ was reacted with 8‐arm PEG to form *native*‐Caf1^RGDS^ hydrogel. Step (ii). This hydrogel was melted, immediately cooled to room temperature and a suspension of hDFb added. After cooling, *refolded*‐Caf1^RGDS^ was formed encapsulating hDFbs. c) Step (i). *Native*‐Caf1^WT^ and native‐Caf1^RGDS^ was reacted with 8‐arm PEG to form *native*‐Caf1^WT^‐Caf1^RGDS^ hydrogel. Step (ii). This hydrogel was melted, immediately cooled to room temperature and a suspension of hDFb cells added. After cooling, *refolded*‐Caf1^WT‐RGDS^ was formed encapsulating hDFbs.

*native*‐Caf1^WT^ or *native*‐Caf1^RGDS^ polymers were converted into *native*‐Caf1 hydrogels by crosslinking (**Figure** **2**a,b, step (i)) with a solution of functionalized NHS‐terminated eight‐arm PEG crosslinker, which forms amide bonds with lysine residues within the Caf1 subunits. The insertion of the RGDS motif into the Caf1 subunits did not affect the mechanical properties of the hydrogels.^[^ ^36^ ^]^ By altering the concentrations and ratios of *native*‐Caf1:PEG crosslinker, it is possible to tune the rheological properties of the resulting Caf1 hydrogels. Although a wide variety of hydrogel formulations are available,^[^ ^37^ ^]^ in this study it was decided to work with 3% w/v Caf1 hydrogels as preliminary work indicated it was sufficiently stiff to study cell culture at longer times and it was particularly efficient and quick to regel after melting. Previous work^[^ ^35^ ^]^ also highlighted that hydrogel obtained after one cycle of melting/resetting (we term a Caf1 hydrogel that has been subject to one complete cycle of melting and resetting as *refolded*‐Caf1 hydrogel) possesses different rheological properties (slightly lower stiffness and increased critical strain) in comparison to the original *native*‐Caf1 hydrogel. It was speculated that this distinction most likely arises because of differences in network topologies, with the *refolded*‐Caf1 network having more dangling ends than the more regular *native*‐Caf1 network, and therefore is likely to be relatively more porous. It is important to note that by using the cell encapsulation approach detailed in this work, where hydrogels were melted and then allowed to reform in the presence of cells, the cells were encapsulated inside hydrogel networks of *refolded‐*Caf1.

Another advantageous feature of Caf1 polymers is that is it possible to utilize their meltable feature to prepare^[^ ^35^ ^]^ copolymers with controlled compositions of different subunits. We have shown that controlling the Caf1^WT^/Caf1^RGDS^ subunit compositions within Caf1 copolymers allows the tuning of copolymer bioactivity. Importantly, copolymers displaying both the bioactive Caf1^RGDS^ and the inactive Caf1^WT^ subunits appear to present more beneficial microenvironment for cells than polymers expressing purely Caf1^RGDS^, presumably as the Caf1^WT^‐Caf1^RGDS^ copolymers avoid oversaturating cell signaling, a known problem with biomaterials displaying a high density of RGDS ligands. Caf1^WT^‐ Caf^RGDS^ hydrogels can be prepared^[^ ^35^ ^]^ by cross linking pre‐ prepared copolymer. However, here we simply crosslinked (Figure 2c, step (i)) a 1:1 mixture of purely *native*‐Caf^WT^ and *native*‐Caf1^RGDS^ polymers to afford the *native*‐Caf1^WT^‐Caf1^RGDS^ hydrogel which was melted and reformed to yield a *refolded*‐Caf1^WT^‐Caf1^RGDS^ hydrogel.

### 2.2 Characterization of Hydrogels

All hydrogels were optically transparent, and the viscoelastic properties of the refolded‐Caf1 hydrogels were measured by rheology (Figure S1, Supporting Information). The results indicate that *refolded*‐Caf1 hydrogels display a chemically crosslinked nature, as the storage modulus (G´) is almost independent of frequency. The insertion of RGDS motifs into the Caf1 structure did not change the mechanical properties of the hydrogels, consistent with previous work.^[^ ^38^ ^]^ We have shown previously^[^ ^35^ ^]^ that *refolded*‐Caf1 polymers obtained from a cycle of thermal unfolding/refolding are much shorter in length than native‐Caf1 polymers, and thus hydrogels obtained from *refolded*‐Caf1 have a lower crosslinking density than their *native*‐Caf1 counterparts.

### 2.3 Encapsulation of Live Cells within Caf1 Hydrogels

We chose to encapsulate neonatal human dermal fibroblast cells (hDFBs), anchorage‐dependent cells whose survival requires attachment to a substrate.^[^ ^39^ ^]^ Fibroblasts are the most abundant cell type within all the body's connective tissues.^[^ ^40^, ^41^ ^]^ They are present in very different tissues and organs, e.g., skin or lung, where their primary role is secretion of the components of the extracellular matrix (ECM). Importantly, fibroblasts demonstrate sensitivity to their surrounding microenvironment, making the hydrogel properties crucial to the observed cellular behaviors.^[^ ^42^, ^43^ ^]^ In this work, encapsulation of hDFbs at a selection of cell densities was explored to investigate if cell density affects the effectiveness of repolymerization. To allow benchmarking, the performance of Caf1 hydrogels was compared with Geltrex, a commercially available hydrogel extracted from murine EHS.

To encapsulate hDFbs cells (Figure 2a–c, step (ii)), the *native*‐Caf1 hydrogels were melted by heating at 100 °C for 5 min and the resulting solutions were immediately cooled to room temperature. hDFb suspensions of different densities (2 × 10^5^, 10^6^, or 2 × 10^6^ cells mL^−1^, low, medium and high cell densities, respectively) were added to the melted gel with gentle mixing. Then, 100 µL aliquots of hydrogel‐cell solutions were transferred to a glass bottom 96 well plate and the hydrogel left to reform at room temperature and then transferred to an incubator at 37 °C, 5% CO_2_. In all cases, the reformation of the Caf1 hydrogels was successfully achieved, even at high cell densities, indicating the Caf1 subunits maintain their capacity to effectively refold and repolymerize even in the presence of live cells. All Caf1 hydrogels were optically transparent under microscope and showed a homogenous distribution of the cells into the hydrogels.

### 2.4 Live/Dead Experiments

**Figure 3:**
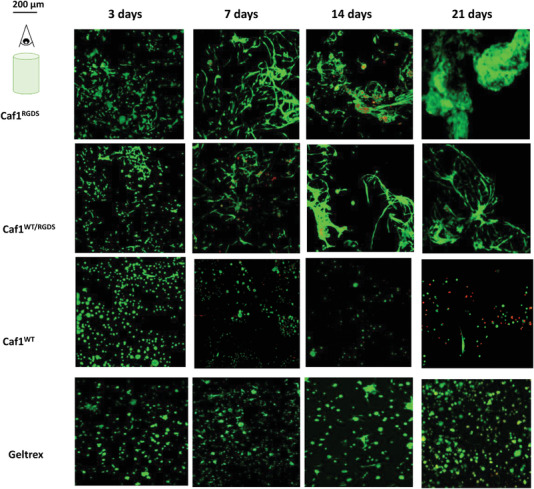
Cellular behavior of hDFbs after hydrogel encapsulation with 10^6^ cells mL^−1^ of hydrogel (intermediate density). Live/dead merged projections of several stack images for cells encapsulated within *refolded*‐Caf1^RGDS^, *refolded*‐Caf1^WT‐RGDS^ and *refolded*‐Caf1^WT^ at 3, 7, 14, and 21 d. Geltrex was used as a control. Live cells were stained with calcein (green color), dead cells were stained with ethidium‐homodimer (red color). The scale bar is shown in top left of the figure so as not to obstruct the images. The images confirmed the excellent cytocompatibility of all hydrogels, with few dead cells observed even at longer times. In the case of cells encapsulated within the *refolded*‐Caf1^WT^ hydrogel, there was a decrement of the number of encapsulated cells with time was observed, most probably on account of the absence of RGDS cell‐adhesion motifs within the hydrogel network.

The viability of the encapsulated cells was tested with live/dead staining experiments after 3, 7, 14, and 21 d, where the samples were incubated for 1 h with calcein to stain live cells (green color) and ethidium‐homodimer (red color) to stain dead cells. Cellular behavior was analyzed by taking z‐stacks images of the hydrogels. For clarity, all these images were merged, and the final picture (represented in 2D) was used. Representative images obtained with the three hydrogel formulations (*refolded*‐Caf1^WT^, *refolded*‐Caf1^RGDS^ and *refolded*‐Caf1^WT‐RGDS^ loaded with a medium cell density (10^6^ cells mL^−1^ of hydrogel)) are shown in **Figure** **3** and Figures S2 and S3 in the Supporting Information. Most of the cells stained green, indicating the cells are alive and that the vast majority of cells successfully survive encapsulation, suggesting strongly the encapsulation method is cytocompatible. All samples showed excellent cytocompatibilities, with most cells alive after 3 d, irrespective of the original density of encapsulated cells. With those hydrogels displaying RGDS motifs (*refolded* ‐Caf1^RGDS^ and *refolded‐*Caf1^WT‐RGDS^), the number of encapsulated cells was observed to increase over the 21 d duration of the experiment. The cells appeared to migrate and proliferate, forming large clusters, suggesting these hydrogels present persistent cytocompatibility over the 21 d course of the experiments and that the hydrogels not only act as a support for the cells, but also provide an environment conducive to proliferation and ECM protein secretion. Conversely, in the case of the *refolded*‐Caf1^WT^ hydrogel, the number of encapsulated cells decreased with time, most probably on account of the absence of cell adhesion ligands within the hydrogel network. The fact that cells can be supported by the hydrogel displaying RGDS motifs, but not by the WT hydrogel, suggests that the absence of cell viability upon the WT hydrogel is simply on account of its nonadhesiveness, rather than any inhibition or toxicity. For cells cultured in Geltrex, the cells appear to display high viabilities even after 21 d, suggesting the material presents good cytocompatibility with hDFbs. However, there was no evidence of formation of the large cell clusters observed with hydrogels featuring Caf1^RGDS^, suggesting that the Caf1 hydrogels promoted cell proliferation and migration more effectively than the Geltrex.

**Figure 4:**
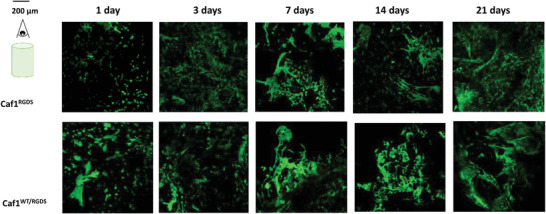
Cellular behavior of hDFbs after hydrogel encapsulation with 2 × 10^6^ cells mL^−1^ of hydrogel (high cell density). Merged projections of live/dead images for cells encapsulated within *refolded*‐Caf1^RGDS^, *refolded*‐Caf1^WT‐RGDS^ at 1, 3, 7, 14, and 21 d (no attempt was made to observe this cell density encapsulated within *refolded*‐Caf1^WT^). The scale bar is shown in top left of the figure so as not to obstruct the images. Live cells were stained with calcein (green color), dead cells were stained with ethidium‐homodimer (red color). The images confirmed the excellent biocompatibilities of these hydrogels, with few dead cells even at longer culture times.

With high density of cell encapsulation (2 × 10^6^ cells mL^−1^ hydrogel), we also include additional time points at 1d (to obtain confidence that cells survive the relatively high density of encapsulation) and 21 d (to obtain data at longer culture times which are often used in, e.g. organoid culture). The cytotoxicity of Caf1 hydrogels featuring the RGDS motif (*refolded*‐Caf1^RGDS^ and *refolded*‐Caf1^WT‐RGD^) was excellent, even at long culture times (21 d) (**Figure** **4**, Figure S4 in the Supporting Information). With both these hydrogels there was an increment in the number of cells with time, however, under these conditions the difference in RGDS density within the hydrogel network did not cause differences in cellular behavior, possibly on account of the high density of cell–cell interactions that are likely to be present.

**Figure 5:**
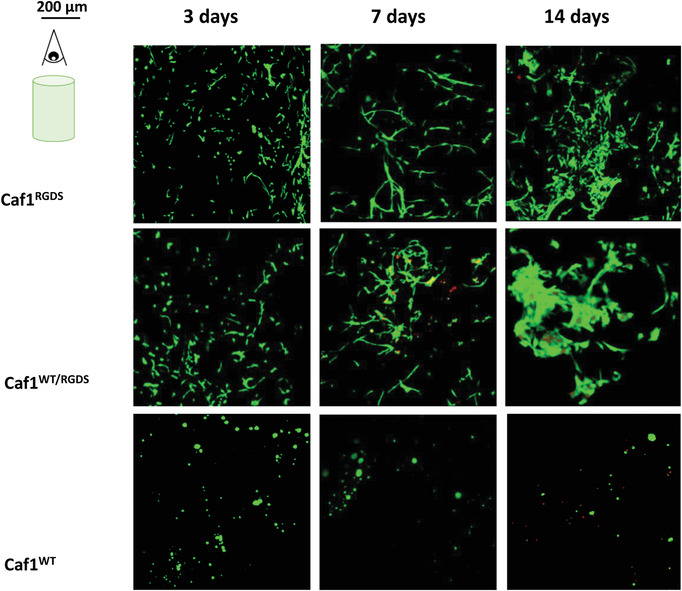
Cellular behavior of hDFbs after hydrogel encapsulation with 2 × 10^5^ cells mL^−1^ of hydrogel (low density). Merged projections of live/dead images for live/dead merged projections of several stack images for *refolded*‐Caf1^RGDS^, *refolded*‐Caf1^WT‐RGDS^ and *refolded*‐Caf1^WT^ at 3, 7, 14, and 21 d. Live cells were stained with calcein (green color), dead cells were stained with ethidium‐homodimer (red color). The scale bar is shown in top left of the figure so as not to obstruct the images. The images confirmed the excellent biocompatibilities of the gels with few dead cells observed even at longer culture times. In the case of *refolded*‐Caf1^WT^, a decrement in the number of cells with time was observed, probably on account of the absence of cell‐adhesion ligands with the *refolded*‐Caf1^WT‐RGDS^ hydrogel, larger cell clusters after 14 d were observed in comparison to the *refolded*‐Caf1^RGDS^ hydrogel, suggesting that the RGDS motif more effectively promotes desirable cell behavior when it is “diluted” with the bioinactive Caf^WT^ subunit.

At the lowest cell density investigated (2 × 10^5^ cells mL^−1^ of hydrogel), the images (**Figure** **5**) showed most of the cells were alive, again confirming the good cytocompatibility of the encapsulation method. Cells encapsulated within the *refolded‐*Caf1^WT^ hydrogel also showed a decrement in the number of cells with time, consistent with experiments at 10^5^ cells mL^−1^ hydrogel. For cells encapsulated within *refolded*‐Caf1^RGDS^ and *refolded*‐Caf1^WT‐RGDS^ hydrogels, an increment in the number of cells with time was observed, however, at this lower cellular density, the increment in the cell aggregation observed in *refolded*‐Caf1^WT‐RGDS^ hydrogel was more significant relative to *refolded*‐Caf1^RGDS^. This observation is likely attributable to the saturation of RGDS motifs in the *refolded*‐Caf1^RGDS^ hydrogel. We speculate that when the RGDS density is decreased through its dilution with Caf1^WT^ subunits, as is the case with *refolded*‐Caf1^WT‐RGDS^, the cellular microenvironment more effectively mimics natural ECM, improving the cellular behaviors. Indeed, several studies have reported^[^ ^44^, ^45^, ^46^, ^47^, ^48^ ^]^ that the optimal behavior of cells was found at specific concentrations of RGDS motifs. We speculate that at the lower cellular density (2 × 10^5^ cells well^−1^) favors cell–matrix interactions, possibly because of the lower number of cell–cell interactions, whereas at higher cell densities cell–cell interactions dominate.

Results of encapsulation experiments at three different cell densities demonstrate that the method of cell encapsulation within Caf1 hydrogels proceeds with high cytocompatibilities, and that hydrogels displaying both Caf1^WT^ and Caf1^RGDS^ subunits within the same hydrogel appear more effective than hydrogels displaying only Caf1^RGDS^. These variations of cell density or RGDS content impacted cell proliferation and clustering when compared with Geltrex, which did not show cell clustering.

### 2.5 Metabolic Activity

The metabolic activity of encapsulated cells was analyzed using the luminescence kit Celltiter Glo 3D (Promega), which determines the cell viability based on the quantification of ATP (adenosine tripho), a marker for metabolic activity. The addition of the reagent results in cell lysis and generation of a luminescent signal proportional to the concentration of ATP, which is directly proportional to the number of viable cells present in culture.^[^ ^49^, ^50^, ^51^ ^]^

**Figure 6:**
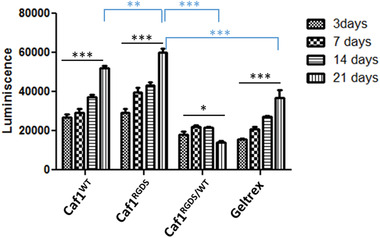
Metabolic activity of encapsulated hDFb cells (10^6^ cells mL^−1^ hydrogel) after 3, 7, 14, or 21 d of culture. Data represent mean values ± SEM (*n* = 3), significant threshold (*p < 0.1, **p < 0.05, ***p < 0.001 and ****p < 0.0001) after two‐way ANOVA with Bonferroni Post hoc test relative to each type of sample. Black stars: significant threshold from day 3 to day 21, same hydrogel. Blue stars: significant threshold comparing at 21 d *refolded*‐Caf1^RGDS^, *refolded*‐Caf1^WT‐RGDS^, and *refolded*‐Caf1^WT^ hydrogels and Geltrex (as control).

The metabolic activity of the cells encapsulated within *refolded*‐Caf1^RGDS^, *refolded*‐Caf1^WT‐RGDS^, and *refolded*‐Caf1^WT^ hydrogels was evaluated at 3, 7, 14, and 21 d, with Geltrex was used as a comparison (**Figure** **6**). For experimental simplicity, cells were encapsulated at a medium cell density (10^6^ cells mL^−1^ of hydrogel) only. With cells encapsulated within *refolded*‐Caf1^RGDS^ and *refolded*‐Caf1^WT‐RGDS^ hydrogels, a significant increment in metabolic activity with time was observed (***p < 0.001), in agreement with the live/dead images. After 21 d of culture, the level of metabolic activity was observed to be significantly higher for cells encapsulated within the *refolded*‐Caf1^WT‐RGDS^ hydrogel in comparison to the *refolded*‐Caf1^RGDS^ hydrogels. This observation is contrary to that previously observed^[^ ^36^ ^]^ by us when hDFbs were cultured in 2D upon Caf1 hydrogels where cells culture upon *refolded*‐Caf1^RGDS^ hydrogels displayed higher metabolic activity in comparison with *refolded*‐Caf1^WT‐RGDS^ hydrogels. It was reported^[^ ^11^ ^]^ that the optimal amount of RGDS in 2D scaffolds is higher than in 3D encapsulation which may explain the differences in cellular behavior observed between 3D and 2D culture.^[^ ^44^, ^45^, ^46^, ^47^, ^48^ ^]^ In 3D cell culture, the optimal amount of RGDS ligands is lower to avoid oversaturating cell signaling, and thus *refolded*‐Caf1^WT‐RGDS^ hydrogels showed better metabolic activity results. Interestingly, the concentration of RGDS ligands in our 3D hydrogels (about 0.05–0.10 mm for *refolded*‐Caf1^WT‐RGDS^ and *refolded*‐Caf1^RGDS^ hydrogels, respectively) are very similar to those estimated^[^ ^52^ ^]^ for natural ECM, a further advantageous feature of our Caf1 platform.

The metabolic activity of the cells encapsulated in *refolded*‐Caf1^WT^ hydrogels was observed to decrease over time, possibly on account of the lack of adherent RGDS ligands, in agreement with live/dead images where a decrement in the number of the cells was also observed.

A significant increase in metabolic activity was observed for cells encapsulated in Geltrex from day 3 to day 21, however, at no time was this metabolic activity higher than that observed for cells encapsulated in *refolded*‐Caf1^RGDS^ or *refolded*‐Caf1^WT‐RGDS^ hydrogels, suggesting Caf1 hydrogels featuring RGDS motifs can mimic the natural ECM more effectively than Geltrex, stimulating cell functions with concomitant increase in cellular metabolic activity.

### 2.6 Immunofluorescence Assays

**Figure 7:**
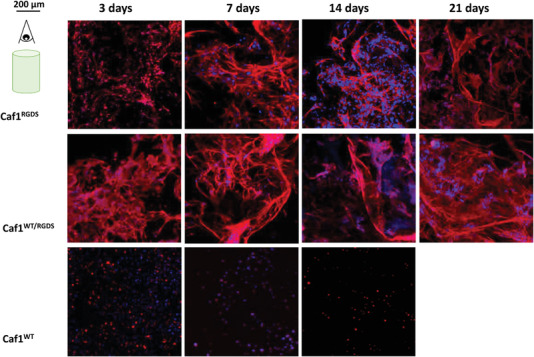
Confocal images of hDFb cells (at a density of 10^6^ cells mL^−1^) encapsulated within Caf1 hydrogels at 3, 7, 14, and 21 d (Red: F‐actin, blue: nuclei). The scale bar is shown in top left of the figure so as not to obstruct the images. On account of the decrease in cell numbers observed with cells cultured within the *refolded*‐Caf1^WT^ hydrogel, the experiment was terminated after 14 d.

We next examined the influence of the hydrogels upon encapsulated cell morphology. Cells were encapsulated inside the hydrogels at a medium cell density (10^6^ cell mL^−1^ hydrogel) and after 3, 7, 14, or 21 d were fixed and their nuclei then stained with 4′,6‐diamidino‐2‐phenylindole (DAPI, blue) and F‐actin stained with Alexa‐Fluor Phalloidin (Red). All the samples were examined at 3, 7, 14, and 21 d to compare cell morphologies and actin filament production (**Figure** **7**). Cellular behavior was analyzed taking z‐stack images of the samples. For clarity, all these images were merged, and the final picture showing a 2D image was used to observed differences. A decrement of cell with time was observed with cells encapsulated within *refolded*‐Caf1^WT^ hydrogels. The cells were also observed to be round and there was no evidence of F‐actin production (filaments stained in red), highlighting how the lack of cell adhesion motifs can affect cellular shape.

For cells encapsulated within *refolded*‐Caf1^RGDS^ and *refolded*‐Caf1^WT‐RGDS^ hydrogels, the production of actin generally appeared to increase with time when images at 21 d are compared with 3d. F‐Actin production, however, is higher in the case of *refolded*‐Caf1^WT‐RGDS^ hydrogel, in agreement with metabolic activity. These results highlight the potential of Caf1 hydrogels displaying RGDS motifs to not only support cells, but also induce cellular bioactivity.

## 3 Conclusions

This work has demonstrated the wide potential of meltable protein hydrogels as artificial ECM for 3D cell culture. Building on our initial findings^[^ ^35^ ^]^ we have here demonstrated that the method is effective over a wide range of cell densities and facilitates the 3D culture of cells for at least 21 d, supporting complex cellular functions such as metabolic activity, cell morphology, proliferation, and migration. The metabolic activity and viability of human dermal fibroblasts within Caf1 hydrogels was modulated by different densities of RGDS motifs, increased with time and, crucially, showed better behavior than commercial animal‐based basement membrane preparations. Since Caf1‐based hydrogels avoid well‐known variability issues associated with animal‐sourced ECM platforms this work is significant because it further highlights the ability of relatively simple, well defined, Caf1‐hydrogels to support complex applications in 3D cell culture.

It is worthwhile discussing briefly issues of potential immunogenicity of Caf1‐based hydrogels. As with any nonhuman protein, Caf1 will provoke an immune response, and this may limit its clinical applications within the body. The preparation of cells for research or in regenerative medicine applications, however, where the protein can be separated from the cells before their ultimate use, are not affected by possible immunogenicity. Much is known about the immunogenicity of Caf1 as it is a *Yersinia* vaccine candidate.^[^ ^53^ ^]^ It does not cause any allergic response when injected into humans and requires the addition of adjuvants and booster injections to illicit a protective immune response, and thus Caf1 is poorly immunogenic in its pure form. Together, these observations indicate that when Caf1 is applied in cell culture applications, there are unlikely to be any issues around immunogenicity.

It is also worthwhile discussing how cells might be released from Caf1 hydrogels. Intriguingly, the actual temperature at which Caf1 unfolds is probably considerably lower than the reported protein melting temperature of ≈86 °C; we estimate its true unfolding temperature to be considerably lower (probably around ≈70 °C); however, unfolding at this lower temperature is incredibly slow (with a half‐life probably in weeks‐months) and still too high to present a viable route to release cells. To address the challenge, we have been actively investigating two strategies: i) the use of low melting temperature mutants and ii) the utilization of redox‐sensitive PEG crosslinkers. We have prepared a Caf1 mutant with an A5I substation in the donor strand which leads to a significant reduction in the protein melting temperature (it will now unfold slowly at ≈45 °C), and we anticipate that hydrogels prepared using a combination of Caf1^WT^ and Caf1^A5I^ subunits will also undergo chain scission at a sufficiently fast rate, which would present a possible route to release unencapsulated cells. We have also been actively investigating the utilization of PEG crosslinkers featuring reducible disulfide linkages, which would allow in principle cleavage of the hydrogel network via the addition of a mild reductant using, e.g., glutathione.

## 4 Experimental Section

### Materials

Succinimidyl glutarate PEG (8‐arm PEG‐SG) (40 kDa) was purchased from Creative PEGWorks (North Carolina, USA). Sodium bicarbonate solution (0.05 M, pH 8.2) was prepared by dissolving of sodium bicarbonate (0.21 g, From Acros) in deionized water (50 mL). The pH was adjusted to 8.2 with dropwise addition of 0.1 M HCl. Geltrex lactose dehydrogenase elevating virus (LDEV)‐free reduced growth factor basement membrane extract, extracted from murine EHS was purchased from Fischer Scientific. DMEM (Dulbecco's Modified Eagle's Medium‐Low glucose D5546) was purchased from Sigma‐Aldrich. Neonatal human dermal fibroblast cells (hDFbs) were purchased from Lonza.

### Protein Expression and Purification

Caf1^WT^ and Caf1^RGDS^ polymers were produced as described previously.^[^ ^38^ ^]^ Briefly, BL21(DE3) *E. coli* cells (New England Biolabs) were transformed with pT7‐COP and pT7‐COP^RGDS^ plasmids, and single colonies used to inoculate Terrific Broth media. The cultures were grown at 35 °C for 22 h before cells were harvested by centrifugation. The supernatant, containing the exported Caf1 polymers, was then passed through a Vivaflow 200, 100 000 MWCO PES tangential flow filtration device (Sartorius) (and the polymers further purified by gel‐filtration using a Capto Core 700 column (Cytiva). To ensure the absence of bacterial contamination, the Ca1 polymers were then sterilized by heating at 65 °C for 15 min in a water bath. To confirm successful sterilization, a sample of sterilized Caf1 material was spread onto an LB agar plate and incubated overnight at 37 °C. No bacterial growth was observed, indicating the success of the sterilization procedure. To quantify endotoxin levels, a Caf1 sample was screened using the PyroGene Recombinant Factor C Endpoint Fluorescent Assay (Lonza) following the manufacturer's instructions. Briefly, Caf1 (100 µg mL^−1^) was incubated with assay buffer, enzyme and chromogenic substrate at 37 °C for 60 min. The fluorescence was measured at 0 and 60 min using Ex/Em wavelengths 380/440 nm and compared to an endotoxin standard curve to obtain a value of 2.90 ± 0.89 EU mL^−1^.

### Hydrogel Formation

Caf1‐hydrogels were prepared by mixing solutions of 8 arm‐PEG‐SG crosslinker (30 mg mL^−1^) in sodium bicarbonate solution (0.1 M, pH 8.2) with Caf1 polymer solutions (3 wt%) at room temperature for at least 1 h.

### Hydrogel Melting and Resetting

Caf1 hydrogels were melted by heating at 100 °C in an oil bath for 5 min. The resulting solution was then immediately cooled in an ice bath at 0 °C for 1 min and was allowed to set at room temperature overnight.

### Rheology Tests

Rheological measurements were performed with an HR‐2 Discovery Hybrid Rheometer (TA Instruments) with standard steel parallel‐plate geometry of 20 mm diameter with a gap of 1 mm. The strain and the frequency were set to 1% and 1 Hz, respectively.

### Cytocompatibility Tests

Neonatal human dermal fibroblast cells (hDFbs) (passages P6‐P9) were cultured at 37 °C and 5% CO_2_ in a humidified incubator using DMEM basal medium, with 10% v/v FBS, 1% penicillin‐streptomycin.

### Cell Encapsulation

Caf1 hydrogels were melted by heating at 100 °C in an oil bath for 5 min and the resulting solution was immediately cooled to room temperature. Cell suspensions of different densities (2 × 10^5^, 10^6^, or 2 × 10^6^ cells mL^−1^ of hydrogel, respectively) were added to the molten hydrogel at room temperature with gentle mixing and then 100 µL of the cell‐containing hydrogel solution was transferred to a glass bottom 96 well plate. The plate was maintained at room temperature for 30 min to allow reformation of the gel and the plate was then transferred to the incubator at 37 °C, 5% CO_2_ and after 4 h, 50 µL of DMEM was added onto the top of gels.

### Live/Dead Staining Assay

Cell viability was assessed by L3224 Live/Dead assay (Life Technologies, UK). In brief, 4 µm ethidium homodimer‐1 and 10 µm calcein (250 µL) in PBS were added to each well and incubated in the dark at 37 °C for 60 min. Then, the samples were washed with PBS and imaged using a Zeiss LSM800 confocal microscope.

### Metabolic Activity

Metabolic activity of the encapsulated cells was investigated using Cell Titer Glo 3D assay (Promega, UK). After the culture period, the culture medium was aspirated and samples were washed with prewarmed DMEM at 37 °C. 50% Cell titer Glo 3D working solution in culture medium was transferred to wells of a 96 well plate. Samples were incubated at 37 °C, 5% CO_2_. Luminescence readings of cell titer solution (100 µL) removed from each well were assessed in triplicate.

### Cellular Morphology Analysis

hDFb morphology and distribution were observed by staining cell cytoskeletons using rhodamine‐phalloidin and the nucleus observed using DAPI. After the culture period, samples were fixed in 4% paraformaldehyde for 60 min and washed three times with PBS. Cells were permeabilized using 0.1% v/v Tween20 in PBS for 10 min. Rhodamine‐phalloidin was prepared using 1:100 dilutions of phalloidin‐tetramethylrhodamine B isothiocyanate (P1951) in 1% v/v Tween20 for 60 min. Residues of rhodamine‐phalloidin were removed by washing samples with 1% v/v Tween20 solution. Samples were immersed in DAPI solution (1 mg mL^−1^) for 30 min and then washed with 0.1% v/v Tween20 in PBS and imaged using a Zeiss LSM800 confocal microscope.

### Statistical Analysis

Data were presented as mean values, standard deviations and/or standard error. Mean values were calculated from three independent experiments of triplicates by group. Statistical analysis was performed by two‐way ANOVA followed by Bonferroni Post hoc tests in GraphPad Prism 5 software using a level of significance of *p* < 0.1 (*), *p* < 0.05 (**), *p* < 0.001 (***) and *p* < 0.0001(****).

## Conflict of Interest

The authors declare no conflict of interest.

## Supporting information

Supporting Information

## Data Availability

The data that support the findings of this study are available from the corresponding author upon reasonable request.
