# Supplementary material for: Exploiting Meltable Protein Hydrogels to Encapsulate and Culture Cells in 3D
Source: Macromol Biosci. 2022 Jul 13;22(9):2200134. doi: 10.1002/mabi.202200134 (PMC11475227; doi:10.1002/mabi.202200134)
Supplement: Supplementary file 1 — Supporting Information [file MABI-22-2200134-s001.pdf]

Macromolecular Bioscience

Supporting Information

for *Macromol. Biosci.*, DOI 10.1002/mabi.202200134

Exploiting Meltable Protein Hydrogels to Encapsulate and Culture Cells in 3D

Gema Dura, Maria Crespo-Cuadrado, Helen Waller, Daniel T. Peters, Ana Ferreira-Duarte, Jeremy H. Lakey and David A. Fulton**

Exploiting meltable hydrogels based on bacterial fimbriae as a method to encapsulate and culture cells within chemically cross-linked hydrogels

Gema Dura,[a,d] Maria Crespo-Cuadrado,[c] Helen Waller,[b] Daniel T. Peters,[b] Ana Ferreira-Duarte,[c] Jeremy H. Lakey,[b] David A. Fulton.[a]

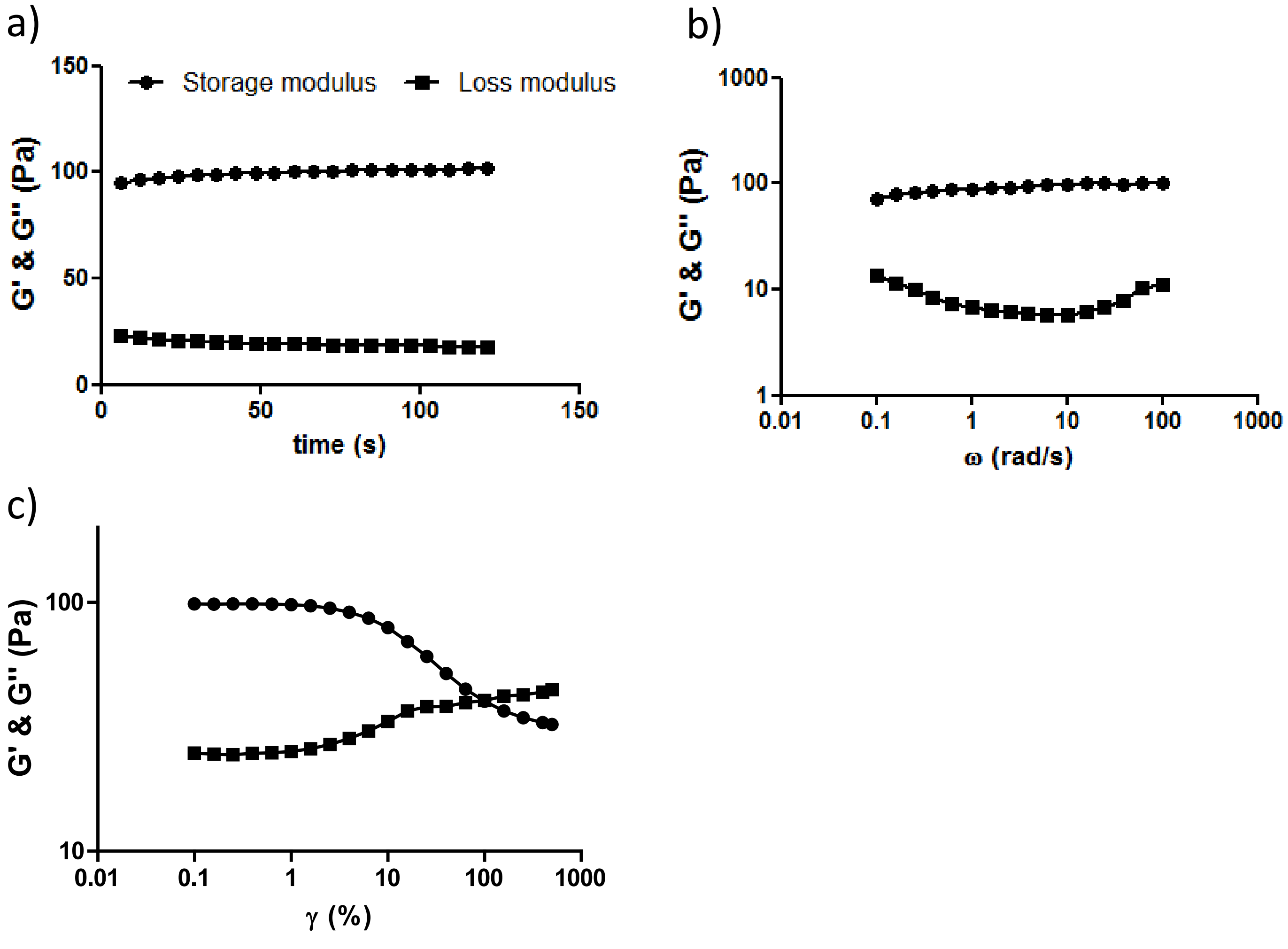

Figure S1: Rheological properties of the gel obtained from refolded-Caf1 hydrogel. a) time sweep experiment of refolded-Caf1WT hydrogel performed at room temperature, at 1 % strain and 1 Hz. b) frequency sweep (ω) experiments performed at room temperature, at 1 % strain. c) Strain sweep (ϒ) experiments performed at room temperature, at 1 Hz.

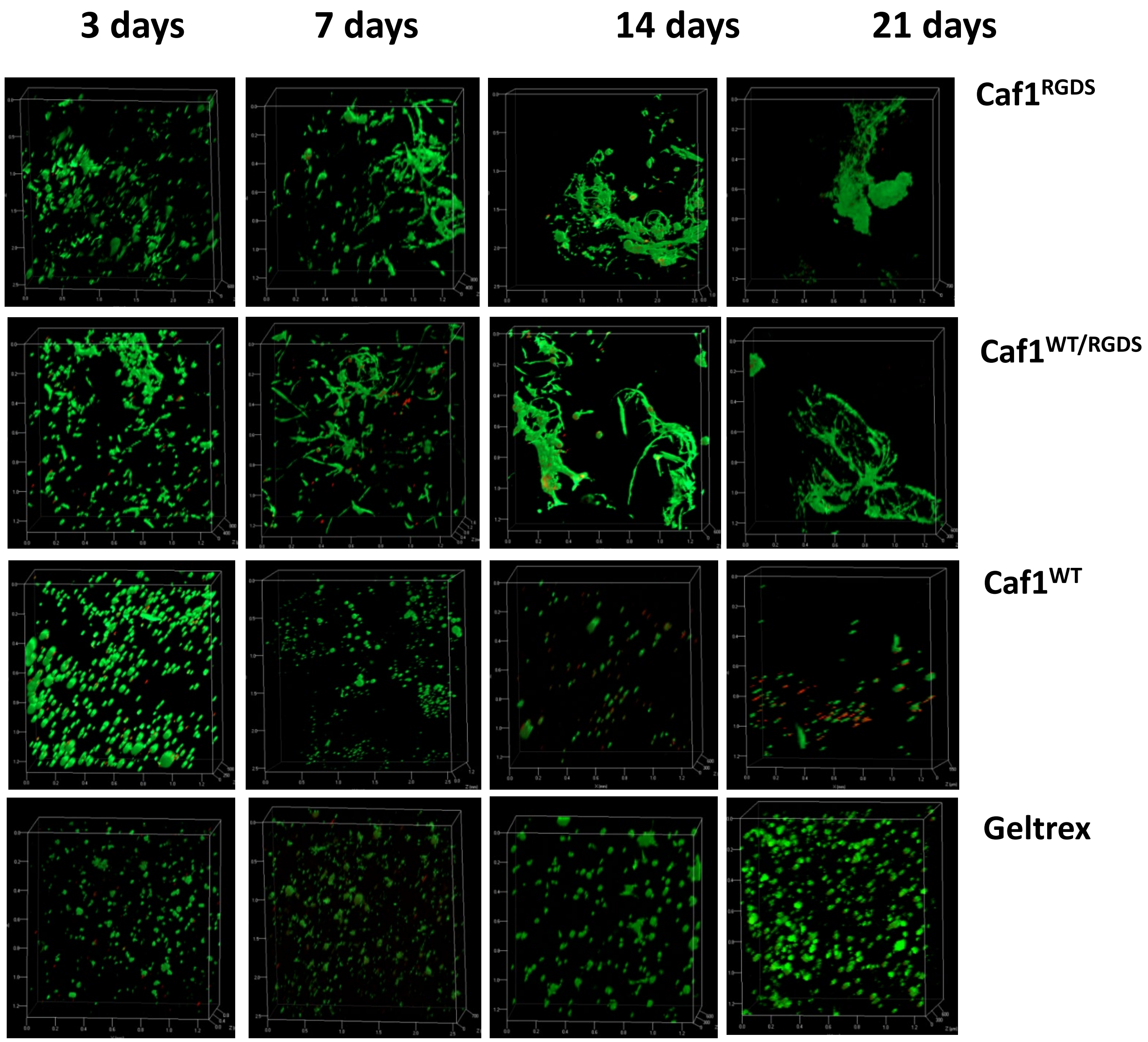

Figure S2: Representation in 3D of confocal laser scanning microscopy revealing the viability of hDFb cells encapsulation in *refolded*-Caf1^{RGDS}, *refolded*-Caf1$^{WT\text{-}RGDS}$ and *refolded*-Caf1^{WT} hydrogels at 3, 7, 14 and 21 d, with 10^{6} cell/mL hydrogel. Live cells are stained in green with calcein-AM and the dead cells with EthD in red.

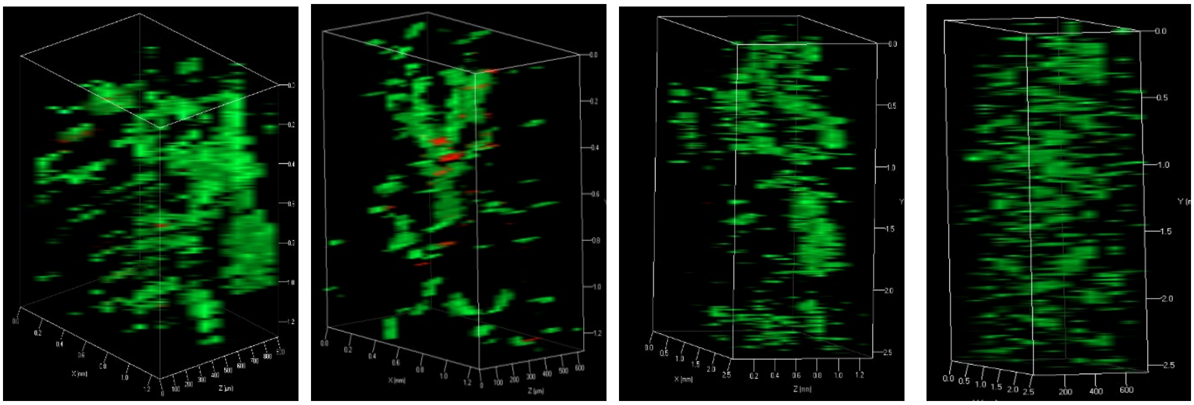

Caf1^{RGDS} **Caf1$^{WT/RGDS}$** **Caf1^{WT}** **Geltrex**

Figure S3: Confocal laser scanning microscopy revealing the cell distribution of hDFb *refolded*-Caf1^{RGDS}, *refolded*-Caf1$^{WT\text{-}RGDS}$ and *refolded*-Caf1^{WT} hydrogels and Geltrex (control) at 7 days, with 10^6 cell/mL hydrogel. Live cells are stained in green with calcein-AM and the dead cells with EthD in red.

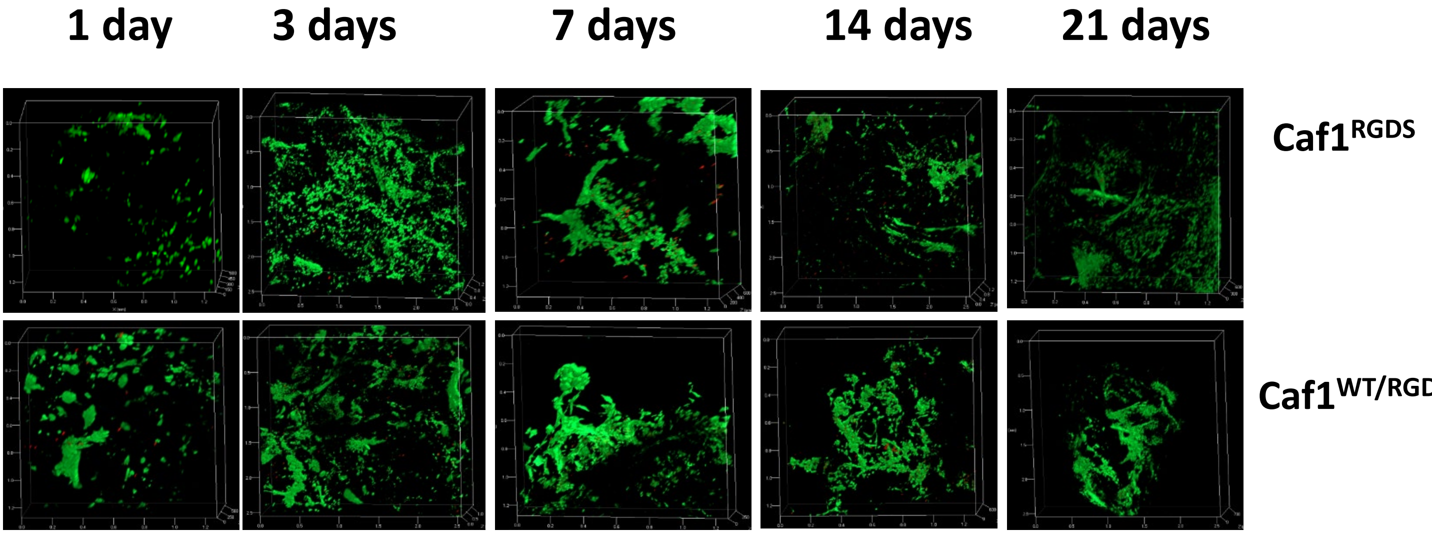

Figure S4: Representation in 3D of confocal laser scanning microscopy revealing the viability of hDFb cells encapsulation in *refolded*-Caf1^{RGDS} and *refolded*-Caf1$^{WT-RGDS}$ hydrogels at 1, 3, 7, 14 and 21 d, with 2 x 10^6 cell/mL hydrogel. Live cells are stained in green with calcein-AM and the dead cells with EthD in red.
